# Relationship between changing cognitive domains and atypical antipsychotic treatment in bipolar disorders: a three-year observational study in a psychiatric rehabilitation center during COVID-19 pandemic

**DOI:** 10.1192/j.eurpsy.2024.1039

**Published:** 2024-08-27

**Authors:** F. Franza, B. Solomita, A. Franza

**Affiliations:** ^1^”Villa dei Pini” Clinic, Psychiatric Rehabilitation Centre, Avellino; ^2^Neamente Study Center, Napoli - Avellino, Italy

## Abstract

**Introduction:**

Bipolar Disorders have been consistently associated with cognitive dysfunction across a broad range of cognitive domains (patients, who usually took psychiatric drugs, sometimes presented changes of cognitive disorders). Many studies have focused on improving the illness severity of patients with MDD or BD by combining mood-stabilizing drugs with atypical antipsychotics (AA). However, the results are contradictory and have not confirmed the certain superiority of AA to other therapeutic strategies. Among these, the cognitive remedy has demonstrated important effectiveness on cognitive variations in this group of patients.

**Objectives:**

In our study, we tried to evaluate some changes in cognitive function in patients with BD treated with antipsychotics related to critical problems with typical cognitive tests.

**Methods:**

In our observational study, we recruited forty-three inpatients (20 females, 23 males) affected by Bipolar Disorder (DSM-5 criteria; particularly 78.5% affected by BD-I), in a psychiatric rehabilitation center. All patients were included in the ordinary rehabilitation treatment. All patients were treated with mood stabilizers (lithium n. 14; valproate n. 29), and at least one AA. The AAs have been the following: quetiapine, aripiprazole, and olanzapine (authorized in Italy)(Table 1). The observation period lasted three years, during three significant waves of the COVID-19 pandemic.

All patients at baseline (T0) (March-April 2020), T1 (Maj-June 2021), T2 (April-Maj 2022), and T3 (April – June 2023) were administered the following rating scales: BPRS, YMRS, GAF, and HAM-D

The data were statistically analyzed with the EZAnalyze 3.0 software for the Excel platform.

**Results:**

In Table 2 and Graphic the results obtained with the rating scales and statistical analysis are shown. In BRPS the data shows a statistically significant reduction in the total score in all periods analyzed. Similar results were found in the GAF and YRMS scales. However, with the HAM-D Scale, there was evidence of an increase in T2, although the differences were not statistically demonstrated. The differences in mean scores are more evident for quetiapine and olanzapine.

**Image:**

**Figure:**
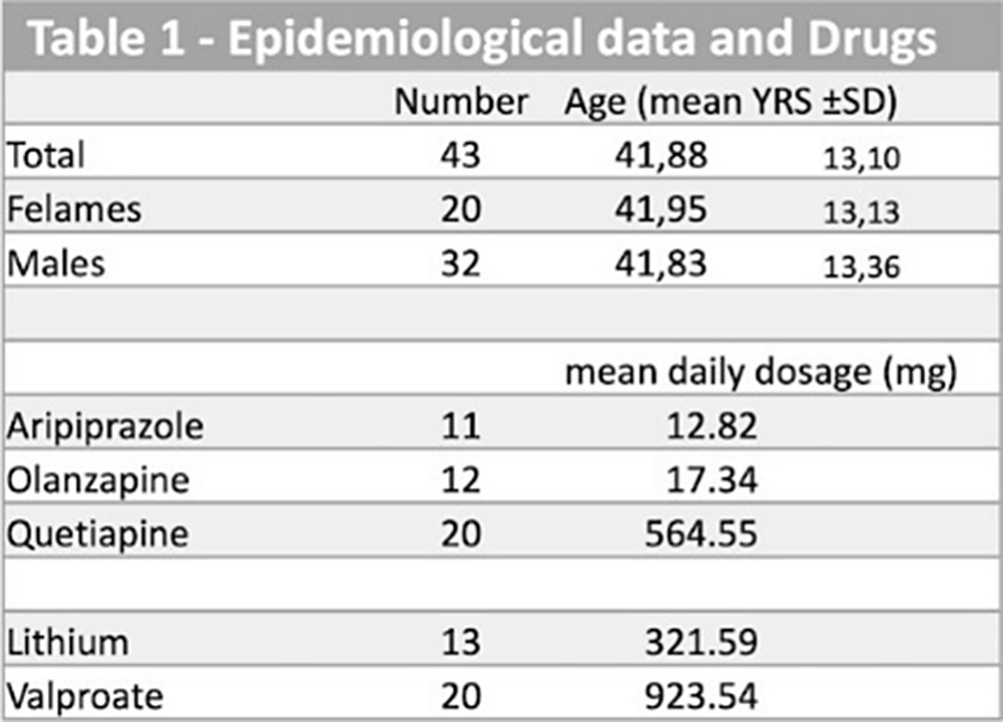

**Image 2:**

**Figure:**
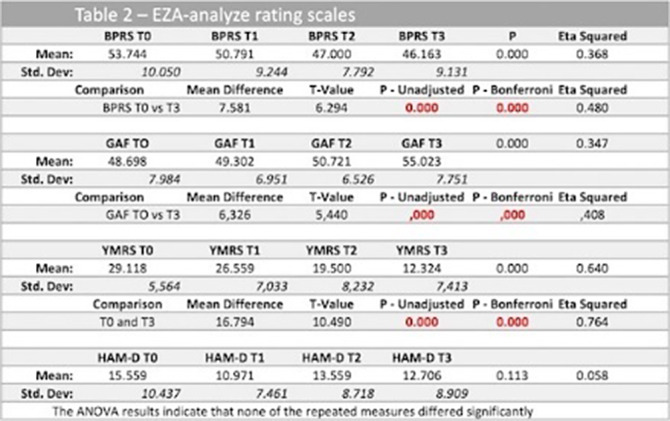

**Image 3:**

**Figure:**
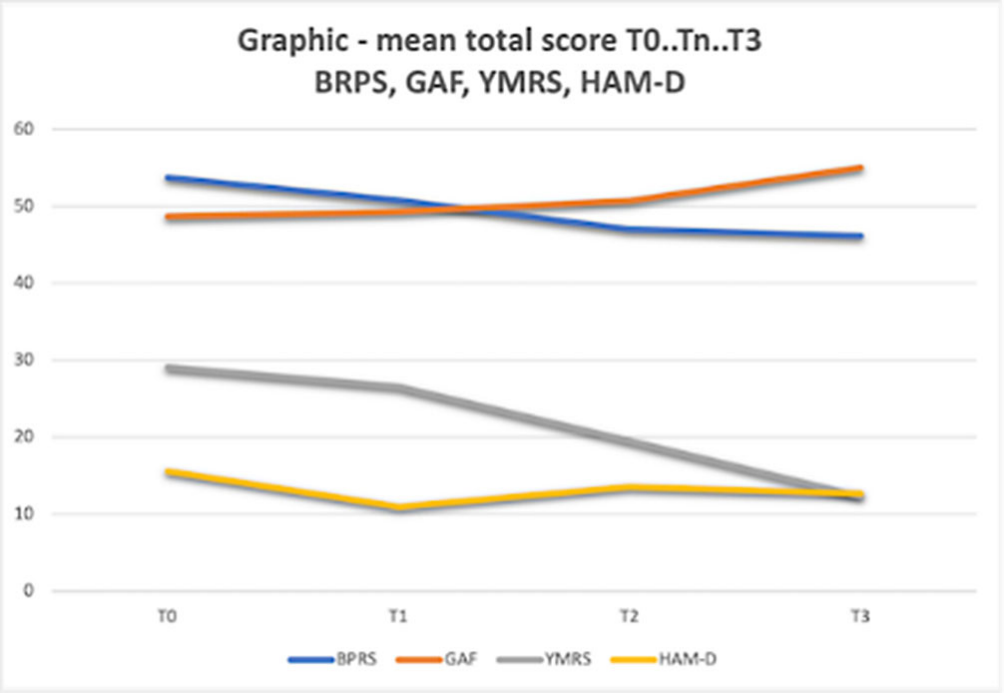

**Conclusions:**

Our observational study showed that the atypical antipsychotics used in our work allowed a significant improvement of the symptoms in BD. However, the pandemic waves have no correlation with the treatment performed. New studies are necessary to highlight the relationship of the pharmacological treatment of BD with the progress of the COVID-19 pandemic.

**Disclosure of Interest:**

None Declared

